# Protocol for laser-induced plasma membrane and cell wall damage in budding yeast

**DOI:** 10.1016/j.xpro.2026.104814

**Published:** 2026-09-02

**Authors:** Yuta Yamazaki, Keiko Kono

**Affiliations:** 1 Okinawa Institute of Science and Technology Graduate University, 1919-1 Tancha, Onna, Okinawa 904-0495, Japan

**Keywords:** Cell Biology, Single Cell, Cell Membrane, Microbiology, Microscopy

## Abstract

Budding yeast is a model organism to study evolutionarily conserved biological processes, including plasma membrane (PM) repair. Here, we present a protocol to induce localized PM and cell wall damage during live-cell imaging. We describe steps for revival from frozen stock, liquid culture, inducing laser damage, and live-cell imaging. We then detail procedures for fluorescence-signal quantification and statistical analysis. This protocol enables the study of localized PM and cell wall repair mechanisms in budding yeast.

## Highlights

• Induction of localized plasma membrane and cell wall damage in budding yeast
• Live-cell imaging of repair-factor recruitment to laser-induced damage sites
• Quantitative analysis of Exo70-NeonGreen recruitment kinetics during repair
• Application to additional fluorescently tagged repair factors in budding yeast

## Abstract (editor highlights)

Publisher’s note: Undertaking any experimental protocol requires adherence to local institutional guidelines for laboratory safety and ethics.

## Graphical abstract

**Figure:**
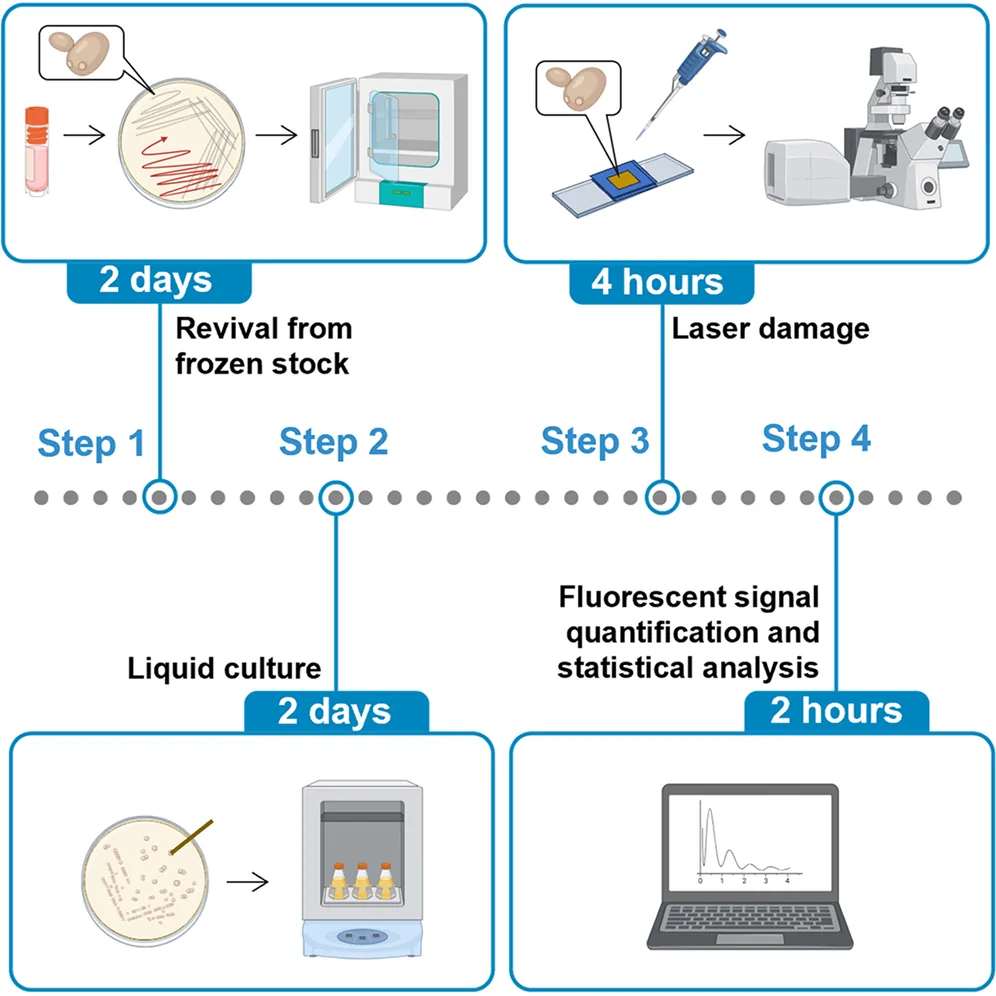

## Before you begin

The fundamental mechanisms underlying plasma membrane (PM) repair are evolutionarily conserved.^1^^,^^2^^,^^3^^,^^4^ Deficits in PM repair are associated with diseases and aging.^5^^,^^6^^,^^7^^,^^8^^,^^9^^,^^10^^,^^11^ However, the mechanisms underlying PM repair remain incompletely understood. Genetically tractable model organisms, including budding yeast, are useful model systems for studying these processes. We present a protocol to induce PM damage in budding yeast to study the PM repair mechanism, using mNeonGreen tagged Exo70 (Exo70-mNG) as a model repair protein. Exo70 is a subunit of the exocyst complex^12^ and likely mediates exocytosis around the damage site.^1^^,^^2^ By combining it with yeast genetics, we have revealed the PM repair mechanisms.^1^^,^^2^ Moreover, we have conducted a proteome-scale screening and comprehensively identified proteins involved in PM repair in budding yeast using this protocol.^3^

### Innovation

We describe a protocol for inducing localized PM and cell wall damage in budding yeast using laser irradiation during live cell imaging. This approach enables controlled, submicron-scale damage to the PM and cell wall in individual yeast cells, allowing quantitative analysis of repair dynamics. Traditional methods for inducing PM and cell wall damage, such as heat shock (37°C–42°C) and sodium dodecyl sulfate (SDS, 0.01%–0.02%) treatment, apply stress to the entire cell surface. Therefore, the methods could not distinguish whether proteins are passively depolarized as a consequence of stress-induced loss of cell polarity, or are actively recruited to and accumulated at the damage site. By contrast, the laser-damage assay presented here induces a single, localized lesion in a submicron-scale region of the yeast cell surface, enabling these two possibilities to be distinguished more convincingly.

### Preparation of coverslip and vinyl-tape chamber for imaging

**Timing: 30 min**

**Figure 1:**
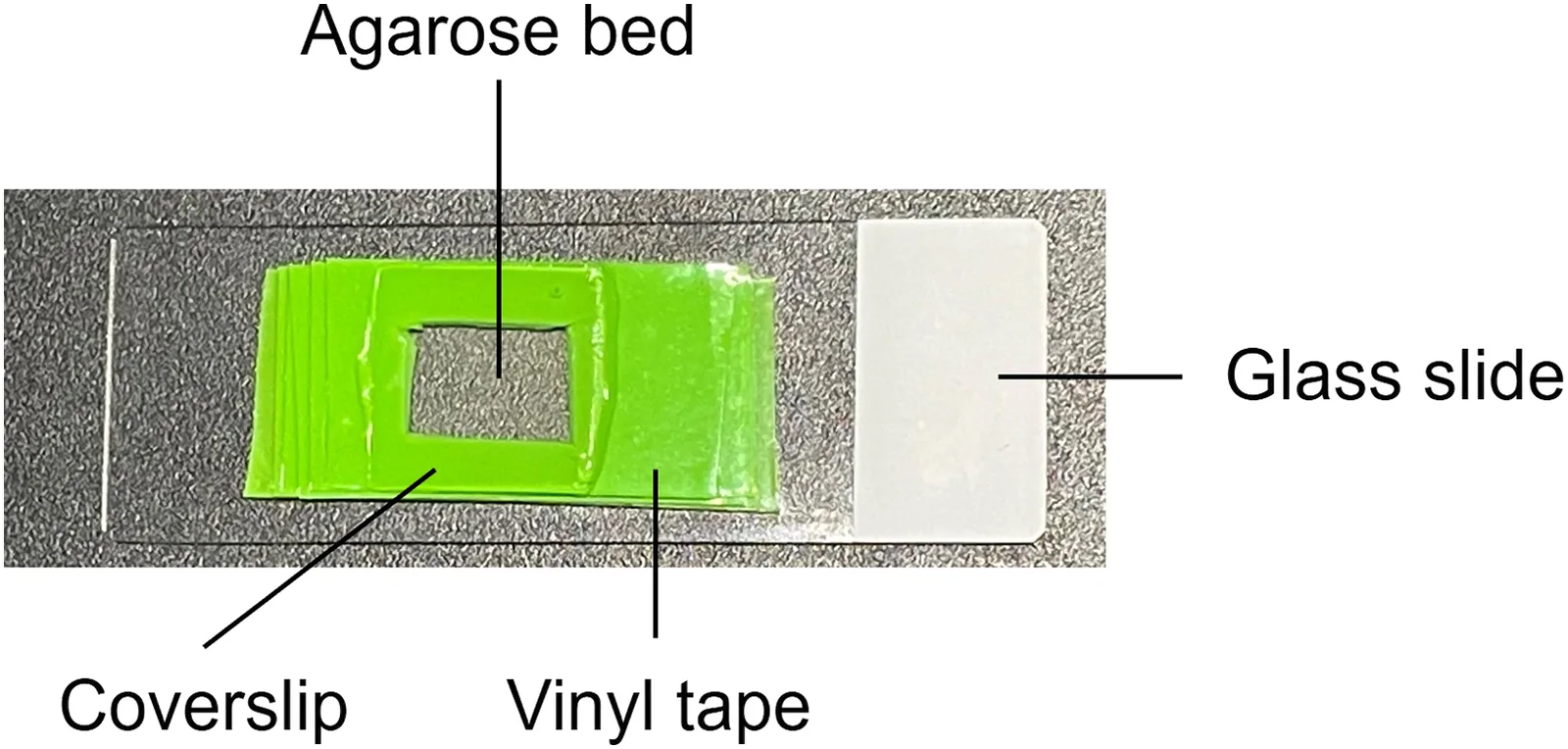
Preparation of a vinyl tape chamber on a glass slide Approximately seven layers of vinyl electrical tape were placed on a NEO No. 1 white-edged glass slide. A central square opening was cut through the tape layers with a box cutter to create a space for the agarose bed (approximately 10 mm × 10 mm × 1.5 mm). Warm SD-Trp agarose medium was then poured into the opening and immediately covered gently with a coverslip. Some agarose may extend beyond the edge of the coverslip; this is acceptable. Once the agarose has cooled to room temperature and solidified, gently remove the excess agarose.

Prepare a vinyl tape chamber using a glass slide (76 × 26 mm) and a cover slip (Dimensions: 18 × 18 mm, Thickness: 0.13–0.17 mm) coated with Concanavalin A (Figure 1).

1. Clean a glass slide with 70% ethanol and Kimwipe. Let it dry on bench for several seconds.
2. Cut vinyl electrical tape (PVC electrical insulating tape; e.g., Scotch Super 33+ Vinyl Electrical Tape, 3M, or equivalent) to ∼3 cm.

***Note:*** Approximately seven layers of vinyl electrical tape are placed on a NEO No. 1 white-edged glass slide.

3. Cut a central square opening through the tape layers with a box cutter to create a space for the agarose bed (approximately 10 mm × 10 mm × 1.5 mm).
4. To prepare agarose bed, add 2.2% agarose to SD-Trp medium and warm in the microwave until the agarose is completely dissolved.

***Note:*** It typically takes about 15 s (600W).

5. Pour the warmed SD-Trp + 2.2% agarose into the vinyl tape chamber.
6. Press the coverslip (18mm × 18mm, Thickness: 0.13–0.17 mm) on the chamber and let it cool to room temperature.

**CRITICAL:** Prepare the agarose bed on the day of use. The vinyl tape chamber can be washed with water and ethanol, thoroughly dried, and reused several times.

### Coating of coverslip with concanavalin A

**Timing: 5 min**

7. To avoid cell movement, coat the coverslips (18mm × 18mm, Thickness: 0.13–0.17 mm), used for the live-cell imaging, with Concanavalin A (Con A). Apply 5 μL of 1 mg/mL Con A.
8. Let the coverslip dry completely at room temperature.
9. Store the coverslip at room temperature until use.

## Key resources table

**Table:** 

REAGENT or RESOURCE	SOURCE	IDENTIFIER
**Experimental models: Organisms/strains**		

Budding yeast strains (BY4741 background) expressing fluorescently tagged proteins	the National BioResource Project (NBRP)	ncbitaxon:1247190 (wild type/no fluorescent tag)

**Software and algorithms**		

Fiji, Image J	Image J	Version 1.54
Prism	GraphPad	Version 9.4

**Other**		

laser scanning confocal microscope A1R	Nikon Instruments	https://www.oist.jp/equipment/img-l007
A1-DU4-2 4 detector unit with 4 multi-alkali photomultiplier tubes (PMT) and CFI Apochromat TIRF 60XC Oil objective lens	Nikon Instruments	https://www.oist.jp/equipment/img-l007
NEO No. 1 white-edged glass slides	Matsunami Glass	S0313
Coverslip 18x18 mm	Matsunami Glass	2-176-01
Concanavalin A	Sigma-Aldrich	C7642
SD-Trp medium	Home made	N/A
SD-Trp agarose medium	Home made	N/A
Sterile wooden toothpicks (autoclaved)	Amy supplier	N/A
vinyl electrical tape	Amy supplier	N/A

## Materials and equipment

**Table:** Synthetic complete media without tryptophan (SD-Trp)

Reagent	Final concentration	Amount
Yeast Nitrogen Base w/o Amino Acids	6.7 g/L	–
L-methionine	85.6 mg/L	–
adenine sulfate	555 mg/L	–
L-arginine	278 mg/L	–
L-aspartic acid	278 mg/L	–
L-glutamic acid	278 mg/L	–
L-cysteine	278 mg/L	–
L-serine,	278 mg/L	–
glycine	278 mg/L	–
L-asparagine	278 mg/L	–
L-alanine	278 mg/L	–
L-Proline	278 mg/L	–
L-glutamine	278 mg/L	–
L-histidine	86.7 mg/L	–
L-isoleucine	278 mg/L	–
L-leucine	520 mg/L	–
L-lysine monohydrochloride	278 mg/L	–
L-phenylalanine	278 mg/L	–
L-threonine	278 mg/L	–
L-tyrosine	278 mg/L	–
uracil	22.4 mg/L	–
L-valine	278 mg/L	–
para-aminobenzoic acid	2.8 mg/L	–
D-glucose	2% (w/v)	–
1N NaOH	Until pH5.6	–

Note on storage conditions e.g., Store at 4°C for up to 2 months.

**Table:** YPD media

Reagent	Final concentration	Amount
yeast extract	1% (w/v)	–
Bacto Peptone	2% (w/v)	–
D-Glucose	2% (w/v)	–

***Note:*** SD-Trip and YPD medium should be autoclaved [121°C 20 min]. D-Glucose should be autoclaved separately as 20% D-Glucose solution.

## Step-by-step method details

### Revival from frozen stock

**Timing: 2 days**

1. Using a sterile toothpick, streak the yeast cells onto the YPD plate from the glycerol stock in −80°C freezer (frozen stocks).
2. Streak the yeast cells onto a YPD agar plate for single-colony isolation, using a fresh sterile toothpick for each streaking step.
3. Incubate the plate without shaking at 25°C for 2–3 days.

### Liquid culture

**Timing: 2 days**

4. Pick a single colony and incubate yeast cells overnight in 5 ml SD-Trp medium in a 25°C water bath until the culture attains an optical density at 600 nm (OD600) of 0.1–0.4.

**Figure 2:**
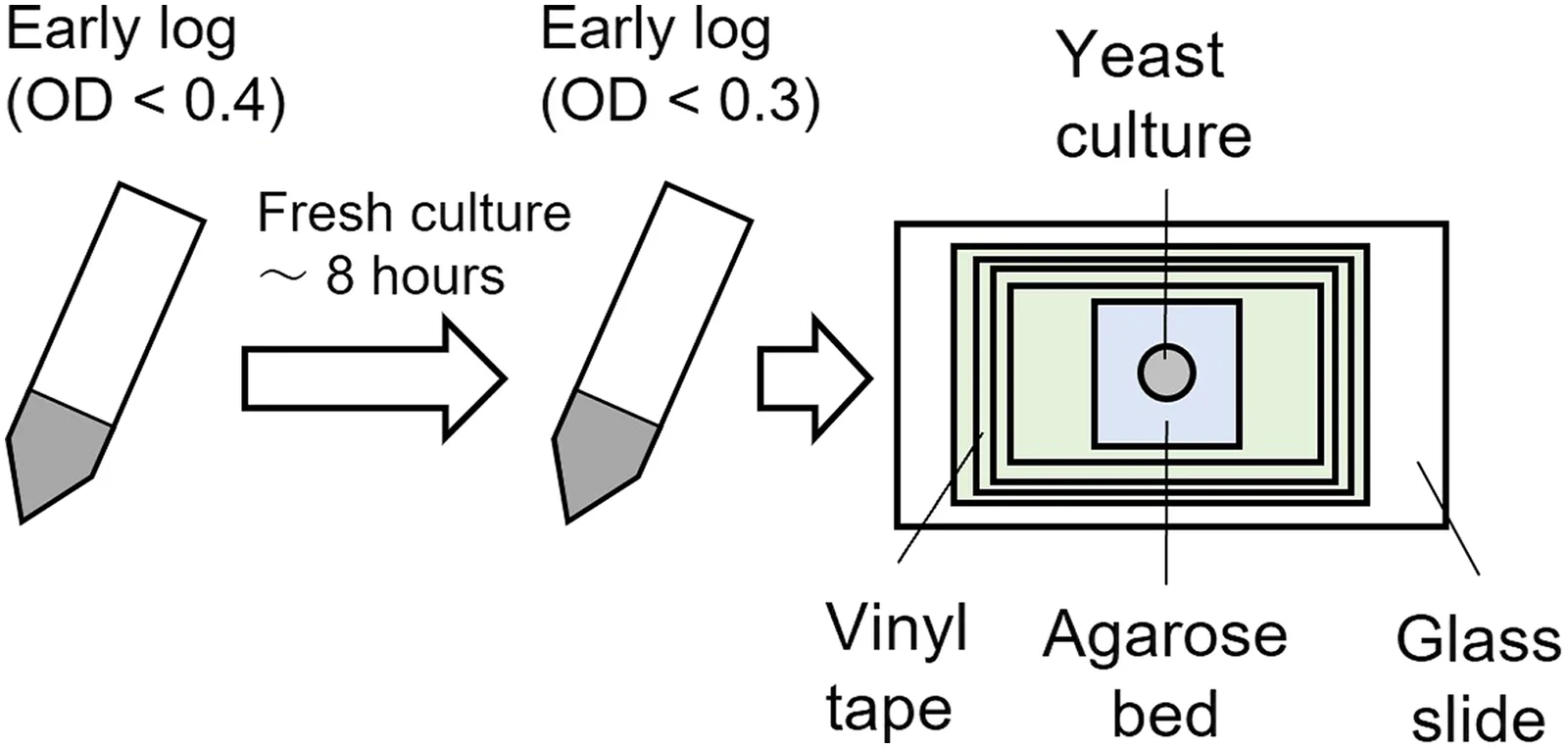
Schematic overview of the procedure Yeast cells are cultured in liquid medium, diluted in fresh medium, and then spotted onto the center of the agarose bed. A coverslip should be placed slowly and carefully over the sample to avoid introducing air bubbles.

***Note:*** YPD medium should not be used due to its high autofluorescence (Figure 2). Although SD medium can be used, SD-Trp medium provides superior fluorescence signals due to its lower autofluorescence.

5. Dilute the yeast culture to OD600 = 0.02–0.05 and further incubate yeast cells in 5 ml SD-Trp media for 3–8 hours at 25˚C until OD600 = 0.1–0.3

**CRITICAL:** The OD600 must be consistent in each experiment to ensure reproducibility.

### Mounting yeast onto the vinyl-tape chamber, inducing laser damage, and live-cell imaging

**Timing: 4 h**

6. Take 1 mL of the culture and centrifuge at 500xg for 5 min to pellet the cells.
7. Remove the supernatant.
8. Resuspend the cell pellet in the SD-Trp medium.
9. Remove the first coverslip from the top of the vinyl tape chamber agarose bed.
10. Take 5 μL of the cell suspension and place it onto the top of the vinyl tape chamber agarose bed.
11. Place the new coverslip coated with ConA, ConA side down, on top of the cells.
12. Apply immersion oil onto the 60× objective lens.
13. Adjust focus until cells are clearly visualized in bright-field mode.

***Note:*** The use of an autofocus system (e.g., Nikon Perfect Focus) is recommended to ensure stable imaging of a defined focal plane.

14. Select a cell to observe and use the zoom function in the microscope software until a yeast cell is clearly visualized.
15. Excite the cell with a 488 nm laser to detect Exo70 tagged with mNeonGreen (mNG).
16. Adjust the signal intensity and the resolution by altering the PMT detector gain, laser power, and/or scanning speed.

***Note:*** We used Nikon A1R Confocal microscope and its detailed setup is as follows: Laser: 405 nm, Objective: 60×/1.49 Oil ApoTIRF WD 0.12, Detector: ×2 MA PMT, ×2 GaAsp, ×1 T-PMT, Software: NIS 6.01.00 / Win10, Resonant Scanner, Perfect Focus, Stagetop Incubator (https://www.oist.jp/equipment/img-l007).

**Figure 3:**
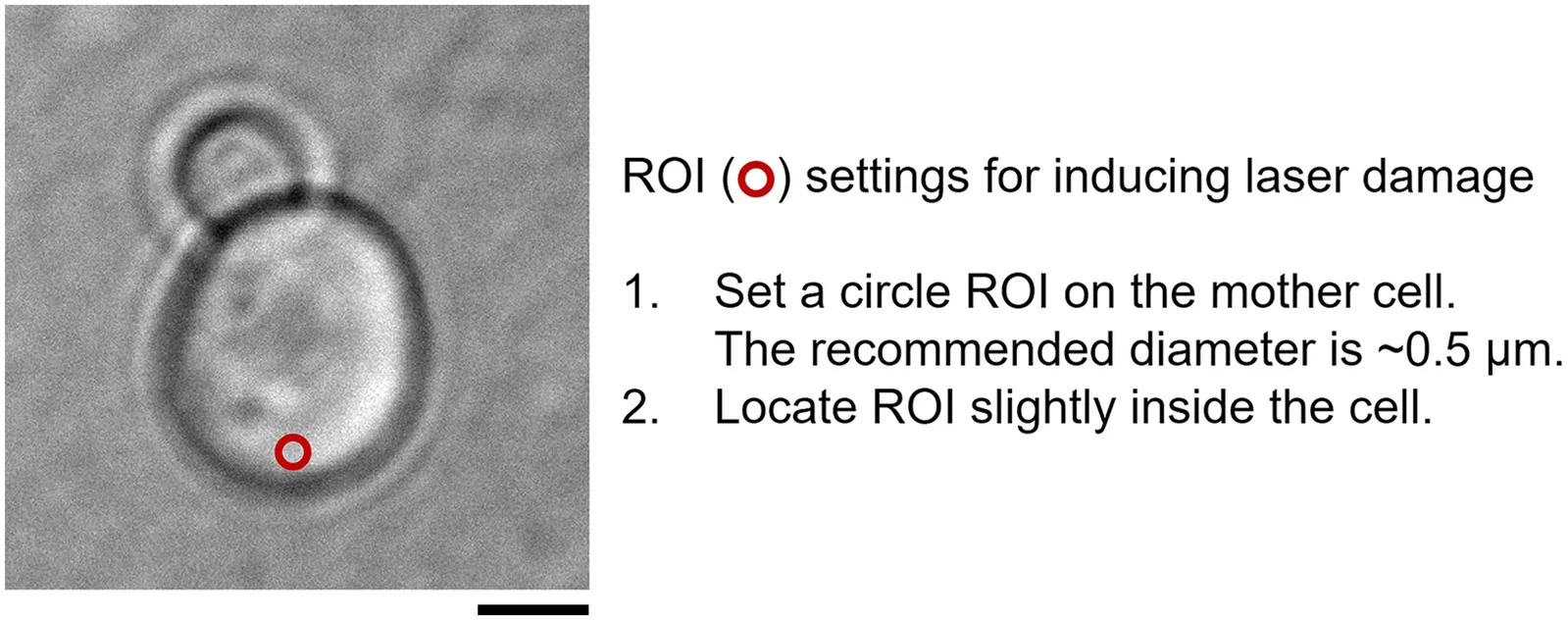
ROI setting for inducing laser damage in budding yeast Scale bar: 2 μm.
18. Optimize the 405 nm laser settings to achieve robust recruitment of Exo70 to the damage sites. **Note:** To do so, use the maximum laser emission setting that does not compromise cell viability. With our microscope system (Nikon A1R Confocal microscope), the optimal laser power setting is ∼50%; the laser energy was 0.7 μJ at the back focal plane of the objective, measured by A Nova II Meter, Ophir Laser Measurement Group.
19. Irradiate cells with the 405 nm laser to induce local damage.
20. Acquire images at appropriate intervals (e.g., 30 sec) until the Exo70 signal appears and ultimately disappears at the damaged site.

***Note:*** Collect 8–15 sets of single focal plane images for each sample.

***Note:*** To minimize potential day-to-day variation, we recommend performing the experiment on three independent days, although we have not observed variation in our experiments.

17. Set the region of interest (ROI) (i.e., the site to be damaged by laser illumination) for optical stimulation with a 405 nm laser (Figure 3).

### Fluorescence-signal quantification and statistical analysis

**Timing: 2 h**

**Figure 4:**
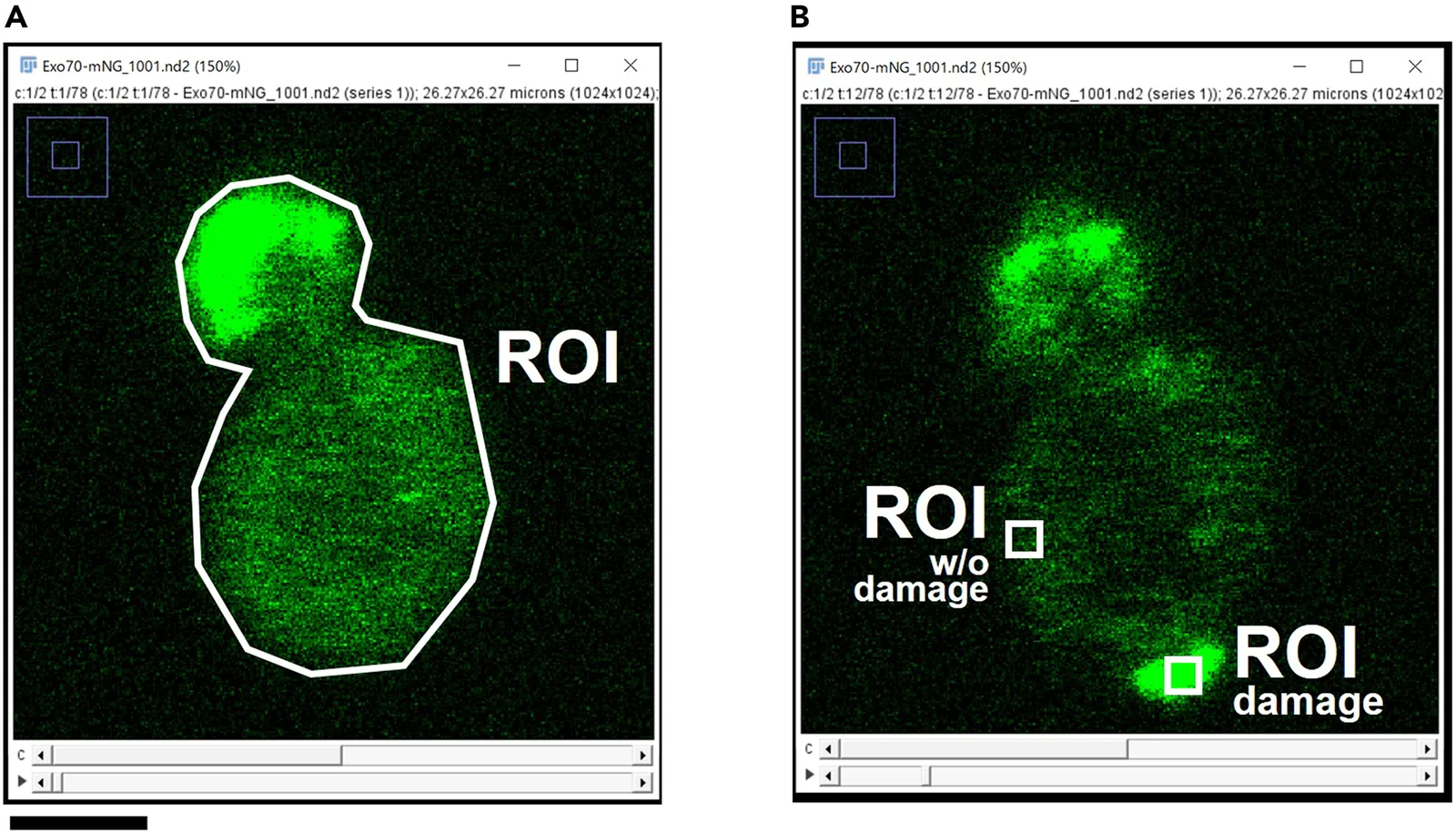
ROI setting for quantifying fluorescence intensity (A) Defining an ROI around an entire cell. Scale bar: 2 μm. (B) Defining an ROI around the damage site and the site without damage.
24. Save ROIs using the ROI manager.
25. Select a ROI around the damage site (Figure 4B).

***Note:*** Since the Exo70-mNeonGreen signal at the damage site frequently moves, we usually set two or three ROIs and use the ROI with the brightest signal as the damage site signal.

21. Open the image file in Fiji software^13^
22. Adjust brightness/contrast until fluorescence is observed.
23. Select a ROI around the entire cell. (Figure 4A).

26. Save ROIs using the ROI manager.
27. Measure the mean pixel intensity of ROIs and export to a comma-separated values (CSV) file using the “Multi Measure” function in Fiji.
28. Calculate the ratio of the mean pixel intensity at the damage site to the mean pixel intensity of the whole cell (Table S1). This is the normalized fluorescence intensity.
29. Plot the normalized fluorescence intensity at the damage site against time after the laser damage.

***Note:*** We used GraphPad prism (version 9.4) and excel for this purpose, although any equivalent software can be used.

30. For comparisons of fluorescence signals between wild-type cells and multiple mutant strains, use Dunnett’s multiple comparison test.

***Note:*** In our analysis, we used Python software (v. 3.11), although any equivalent statistical software or method can be used.

## Expected outcomes

**Figure 5:**
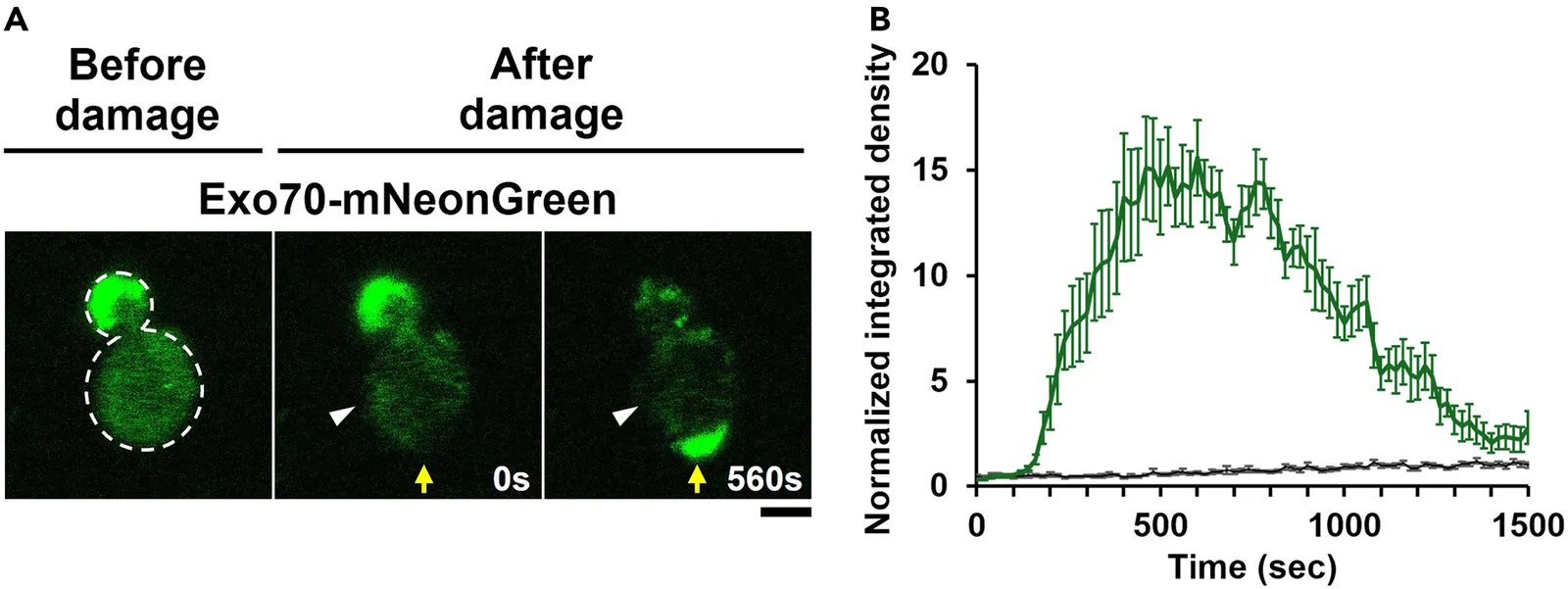
Expected outcome of the laser damage experiments^2^ (A) Representative laser scanning confocal microscopy images of Exo70 tagged with mNeon Green (Exo70-mNG) before and after laser damage. Scale bar: 2 μm. Yellow arrows indicate the site of laser-induced damage. White arrow heads indicate the site without laser-induced damage. (B) The normalized mean integrated density at the damage site over time. Error bars are standard error of the mean (SEM). Green: laser damage site, Black: the site without laser damage.

Expected results are the series of time-lapse images in which Exo70-mNeonGreen or another fluorescence-tagged protein accumulated to the laser damage site. Using 8–10 sets of those images, time-dependent changes of normalized signal intensity at the laser damage site can be quantified and calculated (Figure 5). For reference, example quantification results are shown (Table S1). This analysis allows us to uncover the time course of plasma membrane and cell wall damage response factor (primarily repair factor) accumulation at the laser damage site.

## Limitations

The reproducibility of PM and cell wall damage induction and subsequent repair processes depends on the physiological state of the cells, including growth phase. Therefore, the OD600 must be nearly identical in each experiment. Therefore, reliable and reproducible results can only be obtained with log-phase cells. The size of the laser-induced lesion is sensitive to laser configuration. Therefore, the parameters must be optimized according to each laser and imaging system. Laser output may also be unstable immediately after activation, depending on the laser system used. Allowing sufficient warm-up time can improve the consistency of damage induction. Precise targeting of laser damage depends on the position of the cell within the field of view; cells located near the center of the imaging field often yield the most reliable results. Prolonged live-cell imaging may introduce phototoxicity or photobleaching. For prolonged experiments, preparing a fresh coverslip after approximately 1 h of imaging is recommended to maintain optimal cell conditions. When interpreting the data, care should be taken because laser-induced phototoxicity and photobleaching may affect the apparent repair dynamics. The method described here may also be adapted to other systems, including other fungi and mammalian cells.

## Troubleshooting

### Problem 1

Laser irradiation does not induce detectable damage in the target cell.

### Potential solution

The laser power may be too weak, or the laser irradiation is too short. Increase laser power/irradiation time gradually. In addition, confirm that the focal plane is aligned with the plasma membrane.

### Problem 2

Cells undergo immediate lysis after laser irradiation.

### Potential solution

The laser power may be too strong, or the laser irradiation is too long. Reduce laser power or shorten the irradiation time to induce localized damage without immediate cell lysis.

### Problem 3

Laser damage occurs at a position different from the intended target.

### Potential solution

The target cell may be positioned away from the center of the imaging field, where laser targeting accuracy may decrease. Position the target cell near the center of the field of view before inducing laser damage.

### Problem 4

The extent of laser-induced damage varies between experiments.

### Potential solution

Laser output may be unstable immediately after the laser is turned on. Allow sufficient warm-up time before starting the experiment to stabilize the laser output. Also, there may be differences in physiological state of the cells, including growth phase. Use cultures at similar OD600 and growth conditions to maintain consistent cell physiology.

### Problem 5

Cells show reduced viability or abnormal behavior during prolonged imaging.

### Potential solution

Phototoxicity or photobleaching can happen due to extended live-cell imaging. Reduce excitation intensity and prepare a fresh coverslip after approximately 1 h of imaging.

### Problem 6

The focal plane drifts during time-lapse imaging.

### Potential solution

Mechanical or thermal drift of the microscope stage may happen. Use an autofocus system (e.g., Nikon Perfect Focus) or periodically adjust the focus during imaging.

## Resource availability

### Lead contact

Further information and requests for resources and reagents should be directed to and will be fulfilled by the lead contact, Keiko Kono (keiko.kono@oist.jp).

### Technical contact

For technical specifics on executing the protocol, Yuta Yamazaki (tauyu.kizamaya19940604@gmail.com) will provide support to ensure its correct implementation.

### Materials availability

This study did not generate new, unique reagents or materials.

### Data and code availability

• All data are available from the lead author upon request.
• This article does not report original code.
• Any additional information required to reanalyze the data reported in this article is available from the lead contact upon request.

## Acknowledgments

We thank S. Sugiyama for providing yeast strains and technical advice and Y. Moriyama for advice on the manuscript. A portion of this work was performed with the help of the Okinawa Institute of Science and Technology (OIST) imaging section members. We thank K. Koizumi, T. Mochizuki, S. Komoto, and P. Barzaghi for their technical assistance. This study was supported by 10.13039/501100001700MEXT/10.13039/501100001691JSPS KAKENHI under grant no. 20H03440 and 10.13039/100021022JST-10.13039/501100009023PRESTO JPMJPR1686 to K.K. and JSPS fellowship under grant no. 23KJ2138 to Y.Y. A part of the figures was prepared using bioRender.

## Author contributions

Conceptualization, K.K. and Y.Y.; investigation, Y.Y.; methodology, K.K. and Y.Y.; writing – original draft, Y.Y.; writing – review and editing, K.K. and Y.Y.; supervision, K.K.

## Declaration of interests

The authors declare no competing interests.

## Supplemental information

Table S1. Fluorescence intensity measurements and quantification analyses, related to Step 8
